# The effect of mental accounting in the luxury market: prepared and unprepared consumers

**DOI:** 10.3389/fpsyg.2025.1554350

**Published:** 2025-06-19

**Authors:** Dong Hyun Son, Dongwoo Ko, Ji-yeon Lee

**Affiliations:** ^1^HUFS Business School, Hankuk University of Foreign Studies, Seoul, Republic of Korea; ^2^Department of Counseling Psychology, Graduate School of Education, Hankuk University of Foreign Studies, Seoul, Republic of Korea

**Keywords:** mental accounting, luxury attitude, subjective inequality, SNS usage, process model

## Abstract

**Introduction:**

Social networking sites (SNSs) are increasingly influencing consumer behavior and attitudes toward luxury consumption by fostering social comparison and perceived inequality.

**Methods:**

We conducted a survey with 320 U.S.-based participants through Amazon Mechanical Turk. Measures included SNS usage, subjective inequality, mental accounting, and luxury attitude. Hayes' PROCESS macro (Model 15) was used for moderated mediation analysis.

**Results:**

SNS usage significantly increased luxury attitude directly and indirectly through subjective inequality. Mental accounting moderated both the direct and indirect effects: high mental accounting weakened the direct effect but amplified the indirect effect via inequality.

**Discussion:**

These findings suggest that mental accounting acts both as a self-regulatory buffer and a justification mechanism in luxury consumption. The study highlights the psychological pathway through which SNS usage influences luxury attitude and offers theoretical and practical implications for consumer behavior research.

## 1 Introduction

The widespread availability of internet access contributes to the explosive popularity of Social Network Sites (SNSs) such as Facebook, Twitter, and Instagram. Individuals accustomed to using SNSs are constantly exposed to information posted by others on these platforms, inevitably leading to increased mutual influence among SNS users in their daily lives. The influence of SNSs on individuals is both profound and multifaceted. SNS unquestionably facilitate valuable aspects of modern life, including fostering connections, disseminating information, nurturing online communities and wellbeing (Smith et al., 2020; Wang et al., 2014). On the other hand, the fact that information posted on SNSs tends to present oneself in overly positive ways (Kross et al., 2013; Verduyn et al., 2015) may bring about negative outcomes, such as intensified social comparisons by users and evoking the sense of subjective inequality.

SNS platforms can play a significant role in shaping consumer behavior through social influence (Hyun et al., 2022; Pentina et al., 2018). The impact of SNS usage on the acquisitions and consumptions of luxury products is an evolving topic that has garnered attention from researchers and marketers. As the reach and impact of SNS content continues to expand, SNS users are increasingly exposed to posts and content featuring luxury products and lifestyles (Wang et al., 2021), which can influence their desire to engage in luxury shopping. This increased exposure may influence users to engage in social comparison by observing the luxury purchases of others, especially influencers they admire (Kim and Lee, 2019). Consequently, SNS use can lead to increased luxury consumption and positive attitudes toward luxury products, especially through the mechanisms of social comparison and perceived subjective inequality (Yang and Ha, 2021; Wang et al., 2024; Huang and Zhou, 2024; Wang et al., 2021). Despite growing evidence of this relationship, the links between SNS usage and luxury shopping is not fully explored yet. Many of the existing studies assume that individuals who are exposed to luxury-related contents on SNSs react in a uniform manner. However, this assumption may overlook the importance of psychological and cognitive differences that affect how individuals interpret information acquired via SNSs and make spending decisions. These variances are particularly relevant in the context of luxury goods which are significantly affected by societal perception or economic status. While a number of studies have examined luxury consumption patterns, very few have addressed how financial planning and resource allocation—key aspects of spending behavior—interact with these patterns.

To address this gap, this study introduces mental accounting as a moderating variable in the indirect relationship between SNS use and attitudes toward luxury consumption, mediated by subjective inequality. Mental accounting, one of the concepts in behavioral economics, can play a pivotal role in understanding how people plan for and justify expenditures, particularly in the context of high-cost, non-essential items like luxury goods. Moreover, this study tries to extend the existing knowledge by integrating behavioral economic theory—particularly the notion of mental accounting—into a primarily social-psychological discourse on SNSs and luxury consumption.

From a novel perspective, the study focuses on how individuals' financial cognition, specifically their level of mental accounting, affects their responses to luxury-related content encountered through social media. Mental accounting is a pivotal construct in understanding consumer decisions surrounding luxury goods, as it governs how people allocate and restrict financial resources. Such cognitive mechanisms are likely to influence consumer attitudes both directly and indirectly. By examining the mediating role of subjective inequality and the moderating role of mental accounting, this study provides a deeper and more differentiated understanding of the conditions under which SNS exposure intensifies or weakens luxury consumption attitudes.

## 2 Literature review

### 2.1 SNS usage and luxury attitude headings

Several studies suggest that the use of SNSs has constantly increased with the development of mobile devices and networks, such as 4G and 5G (Duggan and Smith, 2013). The increased popularity of SNS usage provides a whole new opportunity for marketing strategies (Bezawada et al., 2016; Kumar et al., 2022). Through substantial investments, luxury brands such as Louis Vuitton and Chanel are also attempting to increase the exposure of their products on SNS (Phan et al., 2011). Prior literature highlights various benefits of SNS marketing, including stimulating purchase intention and loyalty (Lipsman et al., 2012), fostering customer relationships (Laroche et al., 2013), and improving the credibility of marketing efforts (Goh et al., 2013). Furthermore, Kim and Lee (2019) demonstrate that luxury brands leverage SNS to enhance customer brand attitudes and loyalty. Therefore, it is plausible that attitudes toward luxury brands are becoming more favorable as customers are increasingly exposed to them on SNS.

SNS users can make behavioral changes or consumption decisions not only based on information offered by product companies but also influenced by information provided by other SNS users. Individuals are more inclined to display brands that are consistent with their ideal self rather than their actual self on social media (Wallace et al., 2020). According to Wang et al. (2021), SNS postings related to the consumption of luxury brands can be a powerful external force, motivating SNS users to purchase luxury goods. Also, the use of social media is a significant predictor of materialism, which, in turn, is associated with increased intention to purchase luxury goods (Kamal et al., 2013). These findings suggest that increased usage of social medias leads to the enhancement in luxury attitude. Thus, the first hypothesis is as follow:

*H1: SNS usage enhances the attitude toward luxury*.

### 2.2 Mediating role of subjective inequality on the relation between SNS use and luxury attitude

A unique communication channel provided by SNSs significantly affects the development of social relationships (Eleuteri et al., 2017). With thriving of SNSs, people establish, maintain, and manage their social relationship through not only posting their private information such as pictures, video, music, and so on to SNSs but also reacting to information posted by others. As a result, constant exposure to information on SNSs would increase the possibility of mutually influencing on each other's lives. In other words, there are more chances for SNS users to compare one's life to others' as SNS usage increases (Rice and Hagen, 2010). Moreover, extensive exposure to information on SNS may result in cause the rise of various social problems.

Social comparison theory (Festinger, 1954) indicates that people generally desire to evaluate their capabilities and statuses based on objective criteria. However, under a circumstance where there is no a clear standard, they tend to conduct relative evaluations through comparisons with others around them. Moreover, White et al. (2006) argue that frequent exposures to information about others' activities can lead to increased engagement in social comparisons. According to prior literature (Diener and Fujita, 1997; Diener, 2000), the social comparison is classified into two types: upward comparisons (with superior persons) and downward comparisons (with inferior persons). Accordingly, individuals often using SNSs are more inclined to compare themselves with others because it is relatively easy for them to find out reference groups on SNSs.

As one of major motivations for the use of SNSs is an impression management, contents posted on SNSs predominantly pertain more positive impressions to others, such as enjoying fancy meals, going on outbound travels, or making luxury product purchases (Lin and Utz, 2015). Thus, individuals use SNSs more frequently perceive others as happier than themselves, influenced by the highly biased information shared by their peers (Chou and Edge, 2012). Consequently, individuals on SNSs tend to engage more readily in upward social comparison rather than downward social comparison, leading to an exacerbated sense of subjective inequality which may result in undesirable social consequences such as depress, relative deprivation, and anger.

Economic inequality can be broadly categorized into objective inequality and subjective inequality (Schmalor, 2018). Objective inequality refers to measurable disparities in resources such as income, wealth, or education, typically assessed through large-scale economic or census data (Schmalor, 2018). In contrast, subjective inequality reflects individuals' personal perceptions of being worse off than others, regardless of their actual socioeconomic status (Kelley and Evans, 1993). This perception is often shaped by social comparisons, particularly in environments saturated with selectively positive information, such as social networking sites (SNSs). While both forms of inequality are important, this study specifically focuses on subjective inequality. Our interest lies in the psychological effects of SNS usage, where users are frequently exposed to idealized depictions of others' lifestyles. These repeated exposures can intensify the feeling of being relatively disadvantaged, even among those who are not objectively poor. Therefore, subjective inequality is particularly relevant for understanding the emotional and attitudinal responses—such as resentment or aspiration—toward luxury consumption triggered by social media.

The perception of inequality can influence individual's consumption behavior, such as a positive association between perceived economic inequality and conspicuous consumption, drawing on the “keep up with the Joneses” or “expenditure cascade” hypothesis (Frank, 2007; Christen and Morgan, 2005; Frank et al., 2014). On the contrary, others assert that subjective inequality negatively impacts on conspicuous consumption. present that the extent of economic inequality can impact on the conspicuous consumption, with the increase in economic inequality, wealthier people may reduce spending on conspicuous products because poorer people are required to decrease their conspicuous consumption, influenced by either the income effect or borrowing constraints (Hwang and Lee, 2017). Zabot and Gomes (2022) also provide evidence that conspicuous consumption is negatively associated with economic inequality, especially for credit-constrained individuals. Even if individuals desire to purchase luxury goods for status-seeking, they cannot afford this consumption due to financial constraints, thereby increasing the likelihood that their attitudes toward luxury become significantly negative. Accordingly, SNS users continuously exposed to information related to luxury consumptions on others' SNSs tend to undergo an intensified sense of perceived inequality. This heightened probability, especially among SNS users with a higher level of perceived inequality, will negatively impact on the attitudes toward luxury. As a result, this research investigates whether perceived inequality play a mediating role between SNS use and attitudes toward luxury. The second hypothesis is as follow:

*H2: Subjective inequality mediates the relationship between SNS usage and attitudes toward luxury*.

### 2.3 Mental accounting as a mediated moderator in the association between SNS usage and luxury attitude

The influence of SNS usage and the mediating effect of subjective inequality on attitudes toward luxury may either weaken or become insignificant for some individuals, while strengthening or remaining significant for others. The present study focuses on the individual difference of mental accounting as a possible moderating variable. Thaler (1980) firstly proposes the mental accounting concept developed from a blend of ideas in psychology and microeconomics, defined as “the set of cognitive operations used by individuals and households to organize, evaluate, and keep track of financial activities.” In other words, mental accounting is the cognitive process where individuals group their earnings and expenses into non-fungible mental account categories, allocate money to these categories, set up budgets, and conduct cost-benefit analyses. For instance, individuals allocate their money for foods and travels into separate accounts. In a similar vein, Thaler (1985, 1999) indicates that the same amount of money spent to purchase products for oneself and others (e.g., gifts) is separately considered in different accounts. Previous research documents that such categorization and labeling can influence mental accounting decisions such as spending and other financial decisions (Henderson and Peterson, 1992; Abeler and Marklein, 2017). In addition, categorizing money into various accounts contributes to easing the information processes necessary to evaluate spending opportunities, and such categorization allows individuals to avoid conducting full assessments of their financial assets whenever they are confronted with any consumption decisions, such as the affordability of a certain purchase or appropriateness of resource allocations (Zhang and Sussman, 2017).

Mental accounting not only affects personal spending and saving decisions but also help restrict overspending by imposing budget constraints or limits in specific segregated categories (Shefrin and Thaler, 1988; Heath and Soll, 1996; Muehlbacher and Kirchler, 2019). Mental accounting contributes to controlling expenditures and is particularly beneficial for individuals who lack self-control and self-regulation abilities in financial decisions-making (Mahapatra and Mishra, 2020; Krishnamurthy and Prokopec, 2010). Mental accounting also deters the impulse to indulge in non-essential and unexpected hedonic consumption because such consumption can make individuals feel anticipatory guilt (Cheema and Soman, 2006). Overall, individuals who actively engage in mental accounting are better equipped to exercise financial self-discipline and manage unplanned spending behaviors such as impulsive buying. Although mental accounting can theoretically serve as both a constraint or an enabler in luxury consumption, this study posits that it primarily acts as a constraint in the association between SNS usages and the luxury consumption. This is because the nature of SNS-driven luxury desires often stems from impulsive social comparison rather than rational financial planning (Liu et al., 2019). Therefore, it would be reasonable to consider that the level of engagement in mental accounting may weaken the positive association between the use of SNS and attitudes toward luxury. The third hypothesis is as follow:

*H3: Mental accounting negatively moderates the relationship between SNS usage and the attitude toward luxury*.

As the above literature suggests, mental accounting functions like a double-edged sword—capable of either restraining or reinforcing individual desires, thereby influencing attitudes toward luxury in both favorable and unfavorable ways. Similar to the previous situation, mental accounting can serve as a self-regulatory mechanism that tempers emotionally charged or externally driven consumption impulses, such as impulsive luxury buying. However, under certain circumstances, mental accounting may also play a role as an enabler, positively shaping attitudes toward luxury consumption. Kivetz (1999) highlights that individuals who engage in mental accounting often seek reasons and justifications for indulgent spending, including luxury consumption. Similarly, those with high levels of mental accounting are more likely to require structured rationales and pre-allocated budgets before engaging in luxury purchases (Shefrin and Thaler, 1988; Cheema and Soman, 2006). Accordingly, individuals experiencing heightened senses of subjective inequality may employ mental accounting as a justification and a proper reason for making luxury purchases. These individuals view the acquisition and display of luxury products as enhancing their self-esteem or quality of life (or as a signal of wealth or high social status). By setting aside dedicated budgets for hedonic or luxury expenditures, they establish a financial framework that legitimizes indulgence. In such cases, alleviating the feelings of subjective inequality becomes a compelling motivator for forming mental accounts specifically oriented toward luxury consumption. In other words, among people who experience the feelings of subjective inequality, those who employ the concept of mental accounting may be more likely to construct a detailed financial plan for purchasing or consuming luxuries—provided they have adequate reasons and justifications for doing so. Hence, this study conjectures that the level of individual mental accounting moderates the indirect relationships between the use of SNS and attitudes toward luxury, mediated by the sense of subjective inequality. Specifically, as the level of mental accounting increases, the negative relationship between subjective inequality and attitudes toward luxury is expected to weaken. The fourth hypothesis is as follow:

*H4: Mental accounting moderates the mediating impact of subjective inequality on the relationship between SNS usage and the attitude toward luxury*.

## 3 Methods

### 3.1 Sample and data collection

To assess the hypotheses proposed in this research, we collected data through an internet-based questionnaire administered via Amazon Mechanical Turk (MTurk), a widely used crowdsourcing platform for behavioral research. A non-probability convenience sampling technique was employed. Participants were pre-screened to ensure they were residents of the United States and had prior consumption experience. The survey was conducted over a 7-day period in July 2023, and a total of 320 valid responses were obtained. Among the total respondents, 202 were identified as male (63.1%), and 94.1% identified as White/Caucasian. Table 1 presents the detailed demographic characteristics of the sample.

**Table 1:** Demographic.

**Variables**	**Classification**	**Frequency**	**Percentage (%)**
Gender	Male	202	63.1
	Female	118	36.9
Race	White/Caucasian	301	94.1
	African American	4	1.3
	Hispanic	1	0.3
	Asian	14	4.4
Education level	High school graduate or college level	111	34.7
	College graduate (4 years)	171	53.4
	Postgraduate degree	38	11.9
Age	18–29	69	21.6
	30–39	205	64.1
	40–49	29	9.1
	50–59	11	3.4
	60–69	6	1.9
Income	~$30,000	12	3.8
	$30,001–$60,000	130	40.6
	$60,001–$90,000	135	42.2
	$90,001–$120,000	38	11.9
	$120,001~	5	1.6

### 3.2 Variables

To examine the impact of social networking site (SNS) usage on consumers' sentiments regarding luxury products, an assessment was conducted using a rating scale to measure Active public, active private, and passive Facebook use, along with the Subjective Inequality scale, Mental Accounting, and the Attitude Luxury scale.

#### 3.2.1 Dependent variable

To assess luxury attitudes, the measurement instrument suggested by Dubois et al. (2005) was employed, comprising a total of 32 items categorized into subscales addressing knowledge-related and affect-related themes. Each item in this study was evaluated on a 7-point Likert scale, utilizing the average, and exhibiting a Crombach's alpha of 0.843.

#### 3.2.2 Independent variable

In order to comprehend participants' patterns of SNS usage, adaptations were made to the Active public, active private, and passive Facebook use employed by Frison and Eggermont (2016). The term “Facebook” was adjusted to the more general “SNS” to capture a wider range of SNS influences. The measurement tool comprised subscales for Active public SNS, Active private SNS, and passive SNS, involving a total of seven items. Evaluation was conducted on a 7-point Liker scale, with the mean used for analysis. The Crombach's alpha value was 0.849.

#### 3.2.3 Subjective inequality

To assess the perceived inequality among individuals, we employed the Subjective Inequality Scale introduced by Schmalor (2018). This scale comprises four items designed to gauge inequality, forming one subscale, and an additional four items to assess unfairness, constituting another subscale. The evaluation was conducted through a 7-point Likert scale, and the mean was utilized for analysis (Crombach alpha = 0.912).

#### 3.2.4 Mental accounting

To gauge the influence of Mental Accounting, we utilized a measuring instrument comprising five items recommended by Muehlbacher and Kirchler (2019). A 7-point Likert scale ranging from strongly disagree (1) to strongly agree (7) was employed. The mean value was employed for analysis, and the Crombach alpha value was 0.923.

#### 3.2.5 Control variables

During the data collection phase, an issue emerged with a higher proportion of male participants compared to females. To address potential gender-related effects on luxury products, gender was incorporated as a covariate. Furthermore, recognizing that attitudes toward luxury items can be influenced by economic and social status, the MacArthur Subjective Social Status scale (Adler et al., 1994) was also included as a covariate.

### 3.3 Analysis

To investigate the two hypotheses, we employed Hayes' PROCESS macro version 3.1 (Hayes, 2013). PROCESS Model 15 scrutinizes moderating and mediating relationships, offering bootstrap confidence intervals for both conditional direct and indirect effects. In evaluating H1, which pertains to the direct association between SNS usage and luxury attitudes, and H2, which addresses the indirect pathway mediated by perceived inequality with consideration for mental accounting, we applied the conditional process model. This particular model utilized bootstrap confidence intervals to estimate the conditional direct impact of the independent variable on the dependent variable and the indirect effect through the mediating variable, contingent on the moderator variable.

## 4 Results

The analytical framework utilized was an extensive regression analysis that covered both moderation and moderated mediation aspects. The emphasis was on luxury attitude, where SNS usage served as the primary independent variable, mental accounting functioned as the moderator, and perceived inequality acted as the mediator (Process Model 15; Hayes, 2013; see Table 2). The results show that there was a main effect of SNS usage on Luxury attitude (*B* = 0.935, *p* = 0.001), which support H1. The findings also indicate that with an increase in SNS utilization, there is a corresponding rise in people's perception of the world's unfairness (*B* = 0.948, *p* = 0.001). Also, as the perceived inequality increases, it showed a negative relationship with the luxury attitude (*B* = −0.451, *p* = 0.05). Indirect analysis showed that perceived inequality mediates between SNS usage and luxury attitude (*B* = 0.337, *SE* = 0.078, 95% CI [0.191, 0.494]), which support our proposed H2. Moreover, SNS usage (*B* = 0.935, *p* < 0.001), perceived inequality (*B* = −0.451, *p* = 0.028), and Mental Accounting (*B* = 0.237, *p* = 0.024) were found to be associated with attitudes toward luxury products. Analyzing the interaction terms, a negative correlation is observed between SNS usage and mental accounting with luxury attitudes (*B* = −0.135, *p* = 0.042), while the interaction between inequality and mental accounting demonstrates a positive relationship (*B* = 0.159, *p* = 0.014).

**Table 2:** Results of the conditional process model for luxury intention: the moderation and moderated mediation analysis.

**Variables**	**M (inequality)**			**Y (luxury)**		
	***B***	***SE***	**p-value**	***B***	***SE***	***p*-value**
X (SNS usage)	0.948	0.024	0.000	0.935	0.205	0.000^***^
M (inequality)				−0.451	0.204	0.028^**^
W (mental accounting)				0.237	0.104	0.024^**^
X × W				−0.135	0.042	0.001^***^
M × W				0.159	0.039	0.001^***^
Covariates (ladder)	0.007	0.013	0.574	0.039	0.014	0.006^**^
Covariates (gender)	0.064	0.064	0.315	0.025	0.070	0.723

R^2^ = 0.849, F_(3, 320)_ = 590,428^***^/R^2^ = 0.819, F_(7, 320)_ = 201,466^***^.

Unstandardized regression coefficients, along with their respective standard errors and p-values, are presented. The model controlled for the dependent variable Y (luxury attitude), independent variable X (SNS usage), mediator M (perceived inequality), moderator W (mental accounting), and covariates (gender and social status). The calculations were performed using the PROCESS macro version 3.1.^**^p < 0.01; ^***^p < 0.001.

**Figure 1:**
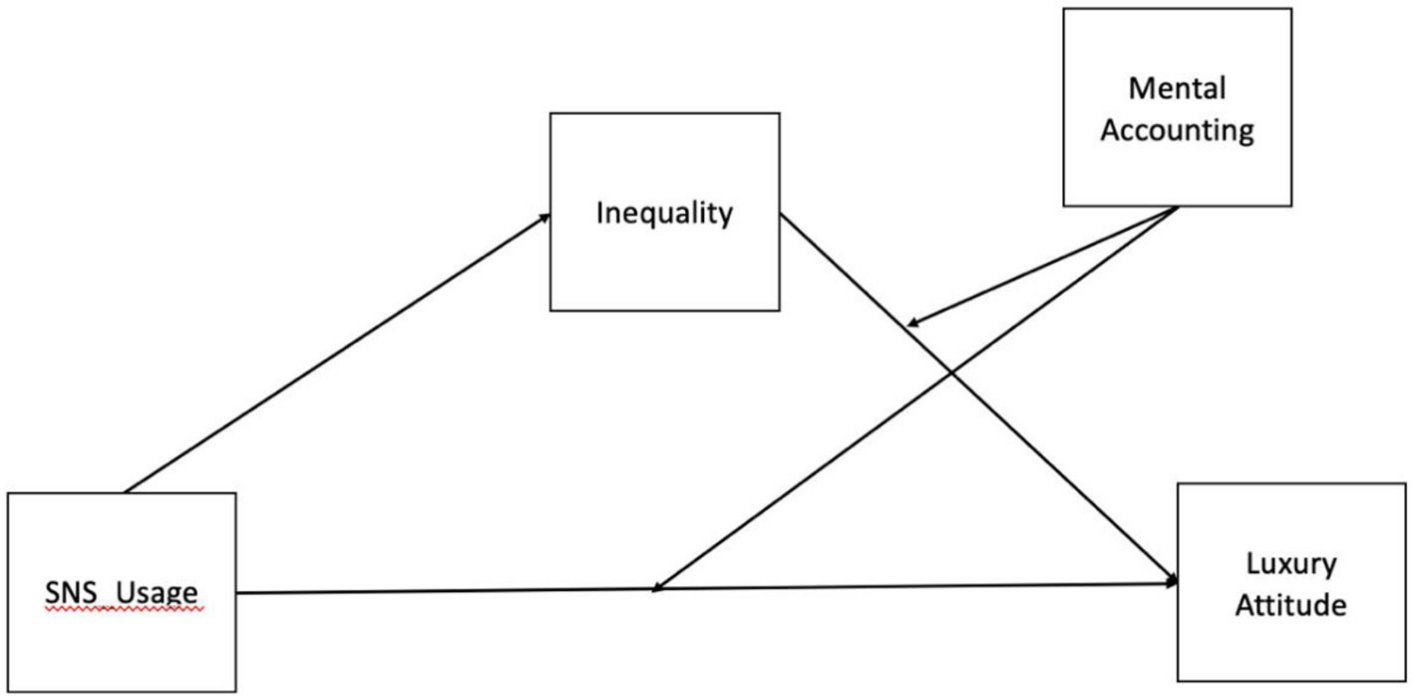
Conceptual model illustrating the hypothesized relationships among SNS usage, subjective inequality, mental accounting, and luxury attitude.

The results from the indirect analysis indicate that the additional conditional process analysis, employing the bootstrapping method, uncovered a dependency of the impact of SNS usage on luxury attitude on mental accounting (refer to Table 3). The conditional direct effect of SNS usage on luxury attitude did not exhibit significance under high mental accounting conditions (*B* = 0.069, *SE* = 0.101, 95% CI [−0.098, 0.235]), but it was significant for both middle (*B* = 0.204, *SE* = 0.075, 95% CI [0.082, 0.328]) and low mental accounting conditions (*B* = 0.529, *SE* = 0.095, 95% CI [0.372, 0.686]). The results support H3 (Figure 1).

**Table 3:** Moderating effect of mental accounting between SNS usage and luxury.

**X → Y**	**Condition**	**Effect (*B*)**	***SE***	***T***	***p*-value**	**95% CI**
	Low mental accounting	0.529	0.095	5.559	0.000^***^	[0.372, 0.686]
	Middle mental accounting	0.204	0.075	2.718	0.007^**^	[0.082, 0.328]
	High mental accounting	0.069	0.101	0.679	0.498	[−0.098, 0.235]

*p* < 0.05 (^*^), *p* < 0.01 (^**^), *p* < 0.001 (^***^).

To assess the impact of perceived inequality on the association between SNS usage and luxury attitude across different mental accounting conditions, we conducted an indirect analysis as presented in Table 4. SNS usage positively influenced luxury attitude (*B* = 0.935, *p* = 0.001), and its effect on luxury attitude remained significantly negative (*B* = −0.451, *p* = 0.028) even after accounting for the control variables. Notably, the interaction term between perceived inequality and mental accounting reached statistical significance (*B* = 0.159, *p* = 0.039).

**Table 4:** Conditional indirect effects of SNS usage on luxury intention through inequality moderated by mental accounting.

**X → M → Y**	**Condition**	**Effect (*B*)**	**Boot *SE***	**95% CI**
	Mental accounting low	0.024	0.205	[−0.293, 0.373]
	Mental accounting middle	0.385	0.098	[0.244, 0.565]
	Mental accounting high	0.535	0.140	[313, 0.779]

The indirect effects revealed that the influence of SNS usage on luxury attitude through perceived inequality was subject to moderation by mental accounting, which supports proposed. Table 4 delineates distinct indirect effects based on mental accounting. The conditional indirect effect of SNS usage on luxury attitude through perceived inequality was significant for both the middle mental accounting condition (*B* = 0.385, *SE* = 0.098, 95% CI [0.244, 0.565]) and the high mental accounting condition (*B* = 0.535, *SE* = 0.140, 95% CI [0.313, 0.779]), but not significant under low mental accounting conditions (*B* = 0.024, *SE* = 0.205, CI [−0.293, 0.373]). Additionally, the results of moderated mediation analysis showed that the indirect effect of SNS usage on luxury attitude via perceived inequality was more pronounced for high mental accounting conditions compared to low mental accounting conditions (*B* = 0.150, Boots *SE* = 0.083, 95% CI [0.003, 0.274]). The results support proposed H4.

**Figure 2:**
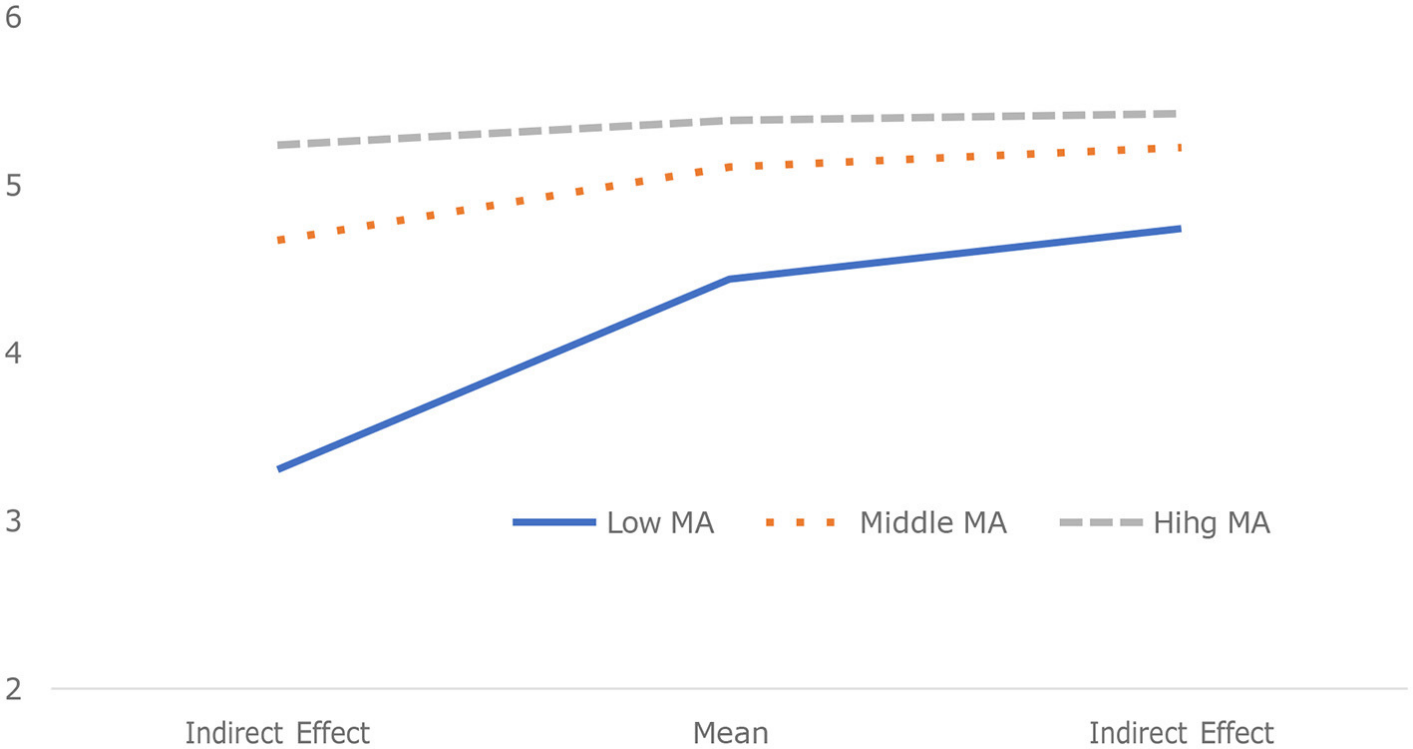
Moderated mediating effect of mental accounting on the indirect effect of inequality.

Further results from the slope analysis illustrated that the slope of the indirect effect was greater under high mental accounting conditions than under low mental accounting conditions, considering the mean and ±1 standard deviation around the mean. This categorization was implemented to enhance the understanding of the moderating effects of mental accounting on the indirect relationship between SNS usage and luxury attitude through perceived inequality (refer to Figure 2).

## 5 Discussion

The study investigated the interplay between social networking site (SNS) usage, luxury attitudes, subjective inequality, and the moderating role of mental accounting in shaping these associations. Especially, incorporating financial planning for purchasing these high-cost items is a noble approach in understanding the role of SNS and luxury attitude. The result of the study confirmed the direct link between SNS usage and attitudes toward luxury (H1), the mediation effect of subjective inequality in the association between SNS usage and attitudes toward luxury (H2), and the moderating effect of mental accounting on both the direct relationship and the mediating impact of subjective inequality on luxury attitudes (H3 & H4). Regarding H1, our findings align with prior research suggesting a significant direct relationship between SNS usage and individuals' attitudes toward luxury (Duggan and Smith, 2013). Participants who engaged more frequently with SNS platforms tended to exhibit a greater inclination toward luxury goods or lifestyles (Lipsman et al., 2012). Users on SNS are inclined to display their ideal self (Wallace et al., 2020), and uploading postings related to the consumption of luxury brands can be a powerful motivation to purchase luxury goods. The result of this study also confirmed the H2 that subjective inequality could act as a mediator in the link between SNS usage and luxury attitudes. According to the social comparison theory (Festinger, 1954), users who spend more time on SNS platforms might be more prone to perceiving disparities in lifestyles, material possessions, or social status among their online connections (Diener, 2000; White et al., 2006). Moving on to H3 to investigate further on factors influencing the associations among SNS usage, luxury attitude, and subjective inequality, mental accounting was investigated together to see how it plays a moderating role in the relationship between SNS usage and attitudes toward luxury, as well as in the mediating impact of subjective inequality on this relationship.

This study's findings hold significant academic and empirical importance in the growing luxury online market alongside the proliferation of social networking services (SNS). Primarily, this research contributes significantly to existing studies on the impact of information exposure on SNS on luxury attitudes. There has been a contradiction between the claim that information related to luxury repeatedly exposed on SNS leads to consumption and the result that it can have a counterproductive effect on consumption. However, this study proposes mental accounting as a factor that bridges these opposing outcomes and verifies its effect.

Individuals with a propensity for mental accounting are more likely to create financial plans for appropriate resource allocations, and individuals with high mental accounting demonstrated a more positive luxury attitude when they perceived subjective inequality through SNS. Meanwhile, individuals with low mental accounting did not exhibit an increased preference for luxury products, even when they perceived subjective deprivation from others' postings on SNS. The study underscores the critical importance of perceiving luxury purchases as viable options, serving as a pivotal factor influencing individuals' luxury attitudes. The findings suggest that the mere willingness to overspend to address perceived deprivation may not be sufficient for a positive association with luxury attitudes. Instead, the viability and feasibility of incorporating luxury purchases into individuals' financial plans play a central role in shaping their overall attitudes toward luxury. This insight challenges conventional perspectives by emphasizing the practicality and financial planning aspects of luxury consumption.

Additionally, the results of this study contribute to the literature on mental accounting. Until now, mental accounting has primarily been studied in financial areas such as investment, perception of price (Siddiqi, 2015), and discounting. However, this research demonstrates that mental accounting acts as an important moderator in understanding people's luxury attitude. Particularly, mental accounting plays a crucial role in understanding the consumption of luxury products, which often involves enduring a high level of financial loss.

### 5.1 Implications

#### 5.1.1 Theoretical implication

This study offers several important theoretical contributions to the literature on consumer behavior, social comparison, and luxury consumption. First, by distinguishing between objective and subjective forms of economic inequality, we reinforce the importance of perceived inequality as a psychological construct. While much of the literature has focused on measurable disparities, our findings highlight the relevance of subjective inequality, particularly in digital environments where social comparison is amplified through social networking sites (SNSs). This extends prior work by integrating the role of SNS-based impression management and social exposure into models of inequality perception and its downstream effects on consumer attitudes.

Second, we contribute to the theoretical understanding of mental accounting by examining its moderating role in SNS-induced luxury consumption. Previous research has documented mental accounting as a cognitive budgeting mechanism, but its interaction with emotional triggers from digital media exposure has been underexplored. Our findings demonstrate that individuals with stronger mental accounting tendencies are less susceptible to SNS-driven luxury desires, suggesting that mental accounting can serve as a psychological buffer against impulsive or symbolically charged consumption. This adds nuance to the theoretical framing of mental accounting, positioning it as a moderator in contexts involving externally driven social and emotional stimuli.

Lastly, this study enriches the literature on luxury consumption by moving beyond trait-level predictors (e.g., materialism, need for uniqueness) and considering situational and emotional mechanisms. By framing luxury attitude formation as a function of social media exposure, perceived inequality, and self-regulatory tendencies, we present a more dynamic, context-sensitive view of consumer psychology in the digital age.

#### 5.1.2 Practical implications

This study also provides meaningful practical implications for marketers, social media strategists, and policymakers. First, the finding that SNS usage heightens feelings of subjective inequality—which in turn can influence luxury consumption—suggests that content design and exposure control on social media platforms are crucial. Brands should be mindful of the psychological effects of curated, idealized content that may unintentionally trigger feelings of inadequacy and reactive consumption behaviors.

Second, our results demonstrate that individuals with high levels of mental accounting are less susceptible to SNS-driven luxury attitudes. This indicates that marketing strategies relying on emotional or impulsive appeals may be less effective for financially disciplined consumers. Instead, marketers should consider framing luxury products as long-term investments or meaningful rewards, which align better with these consumers' budgeting mindset.

Finally, the findings point to the need for segmented marketing communication based on consumers' cognitive and emotional profiles. Rather than applying a one-size-fits-all advertising approach, brands can benefit from tailoring messages to account for differences in perceived inequality and financial decision-making styles. Additionally, platform-level interventions, such as promoting transparency and digital literacy around influencer content, may help mitigate the adverse psychological effects of upward social comparison online.

### 5.2 Limitation

One of the limitations of our study is that we measured participants' luxury attitudes rather than relying on their actual purchase history of luxury products. This choice in measurement might not fully capture individuals' real-world behaviors and engagement with luxury items. While luxury attitudes can provide valuable insights into people's inclinations and preferences, they may not necessarily correlate with their actual purchasing behaviors or financial commitment to luxury products. This limitation underscores the importance of considering both attitudes and behaviors in future research to gain a more comprehensive understanding of the relationship between individuals and luxury goods.

## Data Availability

The raw data supporting the conclusions of this article will be made available by the authors, without undue reservation.
